# Comment on “Symptom phenotypes and coronary microvascular function in non-obstructive coronary artery disease: Insights beyond epicardial ischemia”

**DOI:** 10.1016/j.ahjo.2026.100975

**Published:** 2026-10-16

**Authors:** D. Chamundeeswari, S. Dhanalakshmi, Bhavani Ganapathy, J. Indhuja

**Affiliations:** a Meenakshi College of Pharmacy, Meenakshi Academy of Higher Education and Research, India; b Department of Pharmacology, Meenakshi Ammal Dental College and Hospital, Meenakshi Academy of Higher Education and Research, India; c Meenakshi College of Arts and Science, Meenakshi Academy of Higher Education and Research, India

**Keywords:** Coronary microvascular function, Non-obstructive coronary artery disease, Microvascular dysfunction, Flow reserve, Resistance reserve, Chest pain, Angina, Coronary artery spasm, Fractional flow reserve, Diagnostic accuracy

Dear Editor,

Matsumoto et al. [1], measure flow reserve, index of microcirculatory resistance and microvascular resistance reserve in 59 patients with intermediate left anterior descending stenosis and preserved fractional flow reserve. They relate these measures to atypical and absent chest pain. The question of whether a pressure-corrected reserve index tracks symptoms than the conventional pair is a relevant one. The diagnostic accuracy analysis appears sound. The central descriptive claim in the paper is contradicted by the data it presents.

The abstract and Discussion all state that patients with atypical symptoms had the highest flow reserve and resistance reserve with asymptomatic patients in an intermediate position. The reported medians show the asymptomatic group with values of 3.4 for flow reserve and 4.03 for resistance reserve. The atypical group has values of 3.1 and 3.80. So the asymptomatic group actually has values on both indices, not intermediate. This contradiction appears three times in the paper.

This ordering matters because the severity scale is ordinal. Severity is coded as 0 for no symptoms, 1 for symptoms and 2 and above for Canadian Cardiovascular Society classes. This assumes worsening. The flow reserve values across these categories are 3.4, 3.1 and 2.0. The first step is flat or slightly inverted while the scale treats it as an increase. The correlation of −0.347 and the age-adjusted coefficient of −1.085 thus depend on the versus-everyone-else contrast. One step in the scale is ordered against the data. A two-group comparison would make the point without relying on this assumption.

The diagnostic analysis changes the groups again. Symptomatic is defined as class I or above which includes 21 patients. The 38-patient comparator then includes all 19 patients with chest symptoms. The conclusion that functional dysfunction exists in asymptomatic patients rests on only three of six patients meeting the symptomatic definition. These three may have had chest symptoms.

The resistance null is underpowered not negative. Median values go from 18 to 25 to 30 across the three groups—a rise of two-thirds—at *p* = 0.07 in 59 patients [2]. These values are described as comparable. The paper's conclusion that vasodilatory reserve than minimal resistance reflects symptoms depends on this single *p*-value. The overall median of 25 sits at the structural threshold with 32 of 59 patients above it.

The finding of spasm in the group is a testing artifact. Acetylcholine provocation was done in 13 of 19 patients 2 of 21 with typical angina and none in the asymptomatic group. Spasm occurred where it was sought—in 6 of 13 tested [3]. The discussion treats the between-group difference as meaningful.

Two smaller points: Statin use is reported in 28 patients (47%), but the group counts of 12, 12 and 14 add up to 38 which is 64% [4]. Hyperaemia was induced with either adenosine or intracoronary nicorandil without reporting the split. This matters because resistance reserve includes a correction, for aortic pressure and the two agents affect resistance differently.

The question is worth pursuing. Correcting the group ordering would be the place to start.

## CRediT authorship contribution statement

**D. Chamundeeswari:** Conceptualization, Data curation, Formal analysis. **S. Dhanalakshmi:** Visualization, Writing – original draft, Writing – review & editing. **Bhavani Ganapathy:** Visualization, Writing – original draft, Writing – review & editing. **J. Indhuja:** Visualization, Writing – original draft, Writing – review & editing.

## Informed consent

Not applicable.

## Ethical approval

Not applicable (commentary on a previously published article).

## Declaration of Generative AI and AI-assisted technologies in the writing process

Generative AI tools were used only for language refinement and formatting assistance. All scientific interpretation, critical appraisal, and manuscript preparation were performed independently by the authors.

## Funding

None.

## Declaration of competing interest

The authors declare that there are no conflicts of interest.

## Data Availability

No new data were generated or analyzed in this study.
